# 0042. Burn injury stabilises extracellular atp and induces microvesicle production in skin

**DOI:** 10.1186/2197-425X-2-S1-O11

**Published:** 2014-09-26

**Authors:** U Katbeh, M Takata, KP O'Dea

**Affiliations:** Imperial College London, Section of Anaesthetics, Pain Medicine and Intensive Care, London, UK

## Introduction

Microvesicles (MVs) are subcellular membrane-enclosed particles released from activated or dying cells with multiple functions, including induction of inflammation. A role for MVs has been indicated in sepsis- and trauma-related systemic inflammatory response syndrome (SIRS), but their production and function in severe burns injury is unknown. Extracellular ATP released from mechanically injured cells is an important stimulus for MV production [1], but this response is usually limited spatially as well as temporally by the rapid breakdown of extracellular ATP by ectoATPases. Due to the known sensitivity of ectoATPases to thermal inactivation [2], we hypothesised that ATP released during thermal injury would be stable and capable of inducing MVs from viable responder cells within the burn tissue microenvironment.

## Objectives

To investigate the release of ATP from thermally-injured cells and its potential role in inducing MV release from burn-injured tissue.

## Methods

In vitro cultured human keratinocytes were injured thermally by incubation at 95°C for up to 60 seconds, or mechanically by one freeze-thaw cycle. ATP release was quantified using a fluorimetric assay. An ex vivo mouse skin explant model was developed in which skin pieces were exposed to steam for 15 or 60 secs, or to ATP (6mM) sub-dermally, and then incubated in medium for 2 hours at 37°C. Released MVs were analysed by flow cytometry. Skin oedema was assessed by weight gain during the incubation period.

## Results

**Figure 1:**
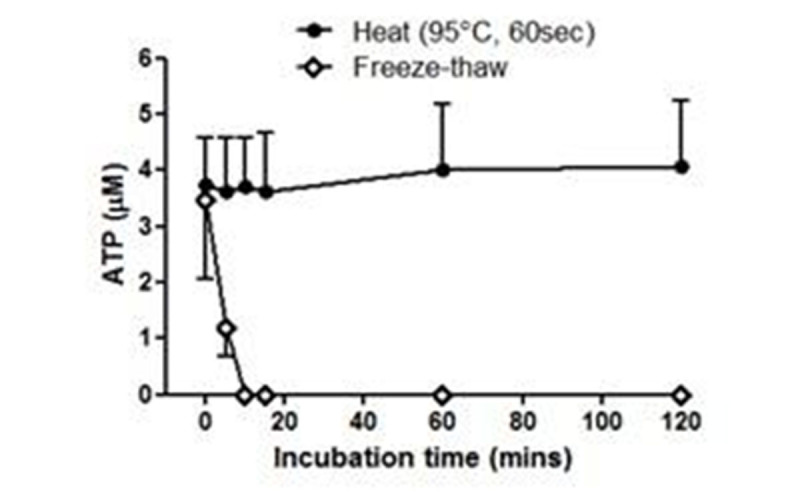
*n*=*4-7*

**Figure 2:**
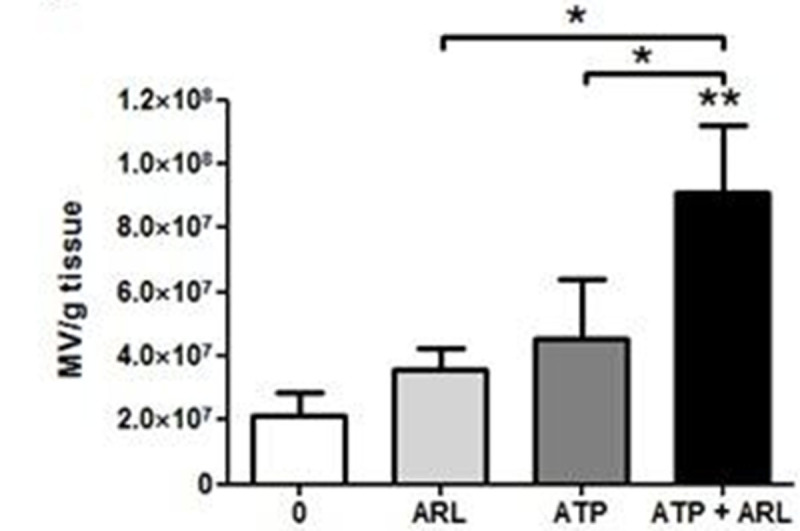
n=4-7, *p<0.05, **p<0.01, ***p<0.001

**Figure 3:**
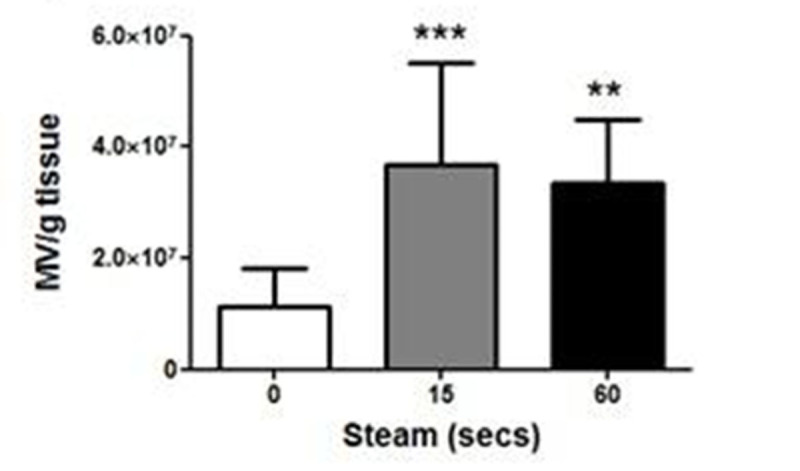
n=4-7, *p<0.05, **p<0.01, ***p<0.001

**Figure 4:**
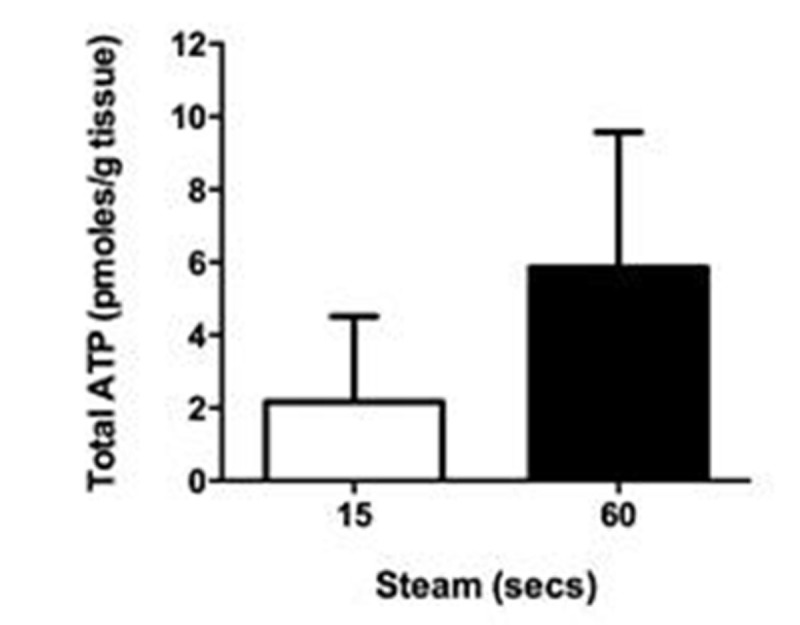
n=4-7, *p<0.05, **p<0.01, ***p<0.001

ATP was released by thermal injury to keratinocytes and remained stable for up to 3 hours at 37°C (Fig. 1). In contrast, ATP released by freeze-thawing decayed rapidly. Incubation of skin explants with ATP produced significant MV release (Fig. 2), but this effect required the presence of an ectoATPase inhibitor (ARL 67156) presumably due to prevention of the normal extracellular ATP degradation process. Exposure of skin to steam for 15 or 60 secs produced significant increases in MV release into the medium (Fig. 3), while ATP was detectable in medium from steam-exposed but not untreated skin (Fig. 4). Skin weight significantly increased with 15 (17.8±4.3%, p< 0.05, n=6) and 60 secs (40.0±18.2%, p< 0.001, n=6) steam exposure confirming that both treatments produced a burn injury.

## Conclusions

These results demonstrate that burn injury can elicit MV production and that ATP release from injured cells could play a central role in this process. The stabilisation of extracellular ATP by burn injury would produce a unique pathophysiological environment for MV production, which, if released systemically, would play an important and more significant role in promoting systemic inflammation following severe burn injury as compared to other SIRS aetiologies.
